# University of Minnesota: Rubric Development for Geriatric Education Programming

**DOI:** 10.1093/geroni/igaf122.1374

**Published:** 2025-12-31

**Authors:** Christina Cauble

**Affiliations:** University of Minnesota, Minneapolis, Minnesota, United States

## Abstract

This presentation focuses on the development and use of a rubric to assess geriatric education products/ outputs such as written work, presentations, or other projects. This presentation focuses on a case example that was used to evaluate team performance in an interprofessional geriatric case competition. The rubric was created to assess key competencies such as teamwork, clinical reasoning, patient-centered care, interprofessional engagement, and application of geriatric principles. Many existing rubrics are generic, pre-designed tools with vague criteria that fail to provide meaningful guidance for students or accurately measure learning outcomes. This highlights the need for geriatric educators to apply evaluative thinking when designing programs and assessments to ensure clarity, relevance, and alignment with learning objectives. The session will cover best practices for rubric development, including ensuring reliability and validity, incorporating stakeholder feedback, and adapting assessment tools for different educational settings. Strategies for piloting, refining, and implementing rubrics effectively will also be discussed, along with common challenges and solutions. By the end of the session, participants will have a deeper understanding of how to create and apply a rubric into one’s geriatric program. They will leave with actionable strategies for developing rubrics that foster transparency, drive continuous improvement, and support meaningful learning in geriatric education.

